# Design and Testing of a Wearable Lower-Limb Exoskeleton for Investigating Falls Prevention

**DOI:** 10.3390/s26144534

**Published:** 2026-07-17

**Authors:** Bethany Gray, Erfan Shahabpoor, Andrew Plummer

**Affiliations:** 1 Department of Architecture and Civil Engineering, University of Bath, Bath BA2 7AY, UK; 2 Department of Mechanical Engineering, University of Bath, Bath BA2 7AY, UK

**Keywords:** human–robot interaction, balance, foot placement, step-length modulation, non-anthropomorphic, under-actuated, partial compensation

## Abstract

Wearable robots that can support balance and prevent falls hold great promise to increase the longevity and quality of life of the older population. However, a lack of understanding of human–robot dynamic interactions and users’ reactions to robot interventions can limit the functionality and usability of these robots. A wearable lower-limb robot was developed to study different strategies to proactively prevent falls during obstacle navigation and to investigate human–robot interactions and users’ reactions to different intervention parameters. A novel non-anthropomorphic architecture was designed for robot legs to allow the direct modulation of foot-placement position in the sagittal plane, using only a single active degree of freedom per leg. Three participants completed a series of walking trials wearing the robot, with different levels of robot intervention. The developed robot was able to successfully modify users’ stride length (e.g., 6–12% and 7.5–20% change in step length for 12 Nm robot hip flexion and extension torques, respectively) in the desired direction, indicating the possibility for assisted balance during obstacle navigation through foot-placement modulation. The measurements show that users’ reactions to the robot intervention is subject-specific and time-varying but, in all cases, plays a considerable role in the final movement trajectory. Controllers of the balance assistance robots must take into account the user’s personalized response to different intervention parameters, to improve functionality, efficiency and user comfort.

## 1. Introduction

Falls are a significant concern for the elderly, with one in three individuals over the age of 65 falling each year in the UK, and the prevalence is increasing [1]. These falls can lead to severe physical injuries such as broken bones, head injuries, and even fatalities. Beyond physical harm, even minor falls can result in substantial mental health issues, including loss of confidence and depression, which can hinder an elderly person’s ability to perform activities of daily living (ADLs) [2,3].

Wearable robots offer a promising solution for fall prevention. However, current devices aimed at balance control and fall prevention are still limited in their functionality and usability. Controllers of fall prevention exoskeletons commonly employ a “reactive” strategy, i.e., detecting an onset of imbalance and then reacting to it to prevent fall. Bayon et al., [4] developed a reactive assistive ankle exoskeleton for unexpected balance perturbations during walking. The robot monitors CoM acceleration to track hip joint movements and provides torque at the stance ankle to counteract these movements. This approach focuses on ankle strategy, but larger perturbations would require hip and stepping strategies to modulate the base of support. A key downside of a reactive strategy is that the torques and velocities required for successful fall prevention, if feasible at all, is likely to be prohibitively high, with substantial functional and safety ramifications [5].

For reliable and safe falls prevention, it is necessary to adopt a more biologically relevant “proactive” strategy, where the possibility of an impending imbalance within the environment is detected and suitable modifications to the movement trajectories are made to pre-empt it. The application of such a strategy to obstacle navigation, however, requires environmental measurements to detect and characterize obstacles’ geometry and characteristics within the user’s path. The lower-limb power assist robot, initially providing flexion/extension assistance at the hip and knee [6] and later adding plantar/dorsiflexion at the ankle [7], uses laser-range finders to detect and characterize obstacles and execute a predefined control scheme. However, it did not take into account the user’s motion plan and their reaction to interventions, necessitating a counter-torque for re-stabilization.

This highlights an important distinction between full-compensation and partial-compensation devices. Users of partial-compensation devices have the autonomy to control their current motion plan, entailing that users’ reactions to robot interventions must be considered within the controller. This is especially true for fall prevention devices, whose intervention may contradict the user’s intended motion plan.

Li et al. [8] developed a controller that detected user instability relative to the support polygon, implementing ankle, hip, or stepping strategies accordingly. However, this assumes that there was no user resistance to the balance strategy. Huynh et al. [9] proposed a reactive balance controller that used the linear inverted pendulum model to determine an “instantaneous capture point” based on CoM parameters. Stability is maintained if the capture point falls within the support polygon. However, without validation of the model with human participants, there were concerns raised about its suitability for able-bodied users. The importance of validating these balance support controllers with able-bodied participants was demonstrated by Zhang et al. [10]. They developed the “Tripping Avoidance Lower Extremity Exoskeleton,” which supported hip, knee, and ankle joints to avoid obstacles. The robot uses impedance control to modify the swing foot trajectory. Despite successful simulations, experimental results revealed inconsistencies due to unconsidered user intentions.

Park et al. [11] used a hip exosuit to explore the effects of hip abduction assistance on walking metabolic cost and balance. The assistive torque, although shown to reduce the metabolic cost of walking in the medial–lateral direction, instigated a full body response and resulted in smaller margin of stability and more unstable walking. Zhu and Yi [12] have used a bilateral knee exoskeleton system for backwards slip prevention. Their results also show changes in whole-body kinematics when subjected to localized knee torque assistance. Afschrift et al. [13] used a bilateral ankle exosuit with a neuromuscular controller to support dynamic walking balance in the anterior–posterior direction after pelvis push–pull, through ankle strategy. Their results have shown a reduction in muscle activity, although this is in contrast with the results of Shafer et al. [14] that showed an increase in soleus activity during early stance and swing and an increase in metabolic cost with a similar controller. The results of all these studies indicate a clear need for improved understanding of human–robot physical interactions and the importance of their integration in the robot controller for an optimal outcome.

With the aim of improving the functionality of balance assistance control models for partial-compensation devices, the wearable robot presented in this paper was designed to enhance our understanding of human–robot interactions during assisted obstacle navigation and proactive trip avoidance. A novel non-anthropomorphic telescopic leg architecture was used to directly modulate foot placement of the swing leg in the sagittal plane without the need for active knee joints.

## 2. Exoskeleton Design

### 2.1. Mechanical Arrangement

The design constraints and requirements of the robot were primarily defined by the biomechanics of foot placement as the target balancing strategy, as well as the wearable nature of the robot. A non-anthropomorphic design was used to (a) minimize the number of active DoFs, size, weight and controller complexity; and (b) ensure accurate measurement of the interaction forces between the robot and the user through minimizing contact points. Using minimal interaction points can further increase usability and comfort.

**Figure 1:**
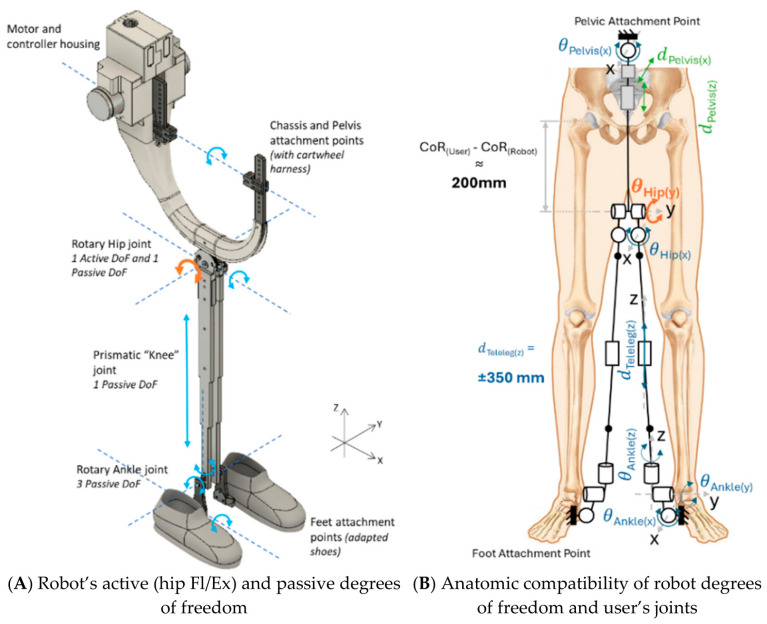

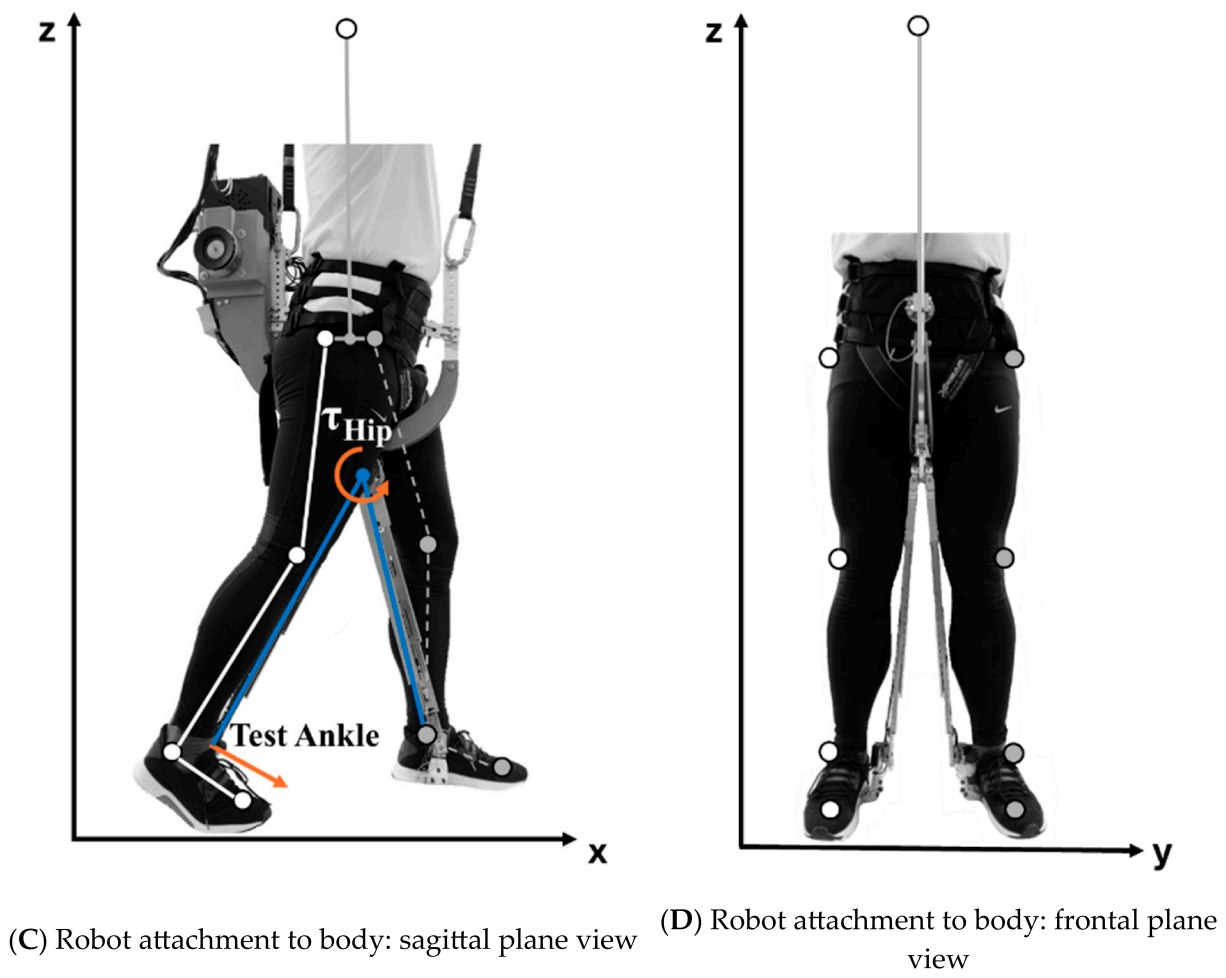
Robot degrees of freedom and attachment to the user’s body.

The use of telescopic legs allowed for only one active DoF per leg to directly control the swing foot-placement position along the walking direction (x-axis—Figure 1). The telescopic legs were connected to the hip joints at the proximal end and to the ankle joints at the distal end. This prismatic joint allowed the hip and ankle joint to be directly connected; effectively bypassing the knee joint. The user’s knee flexion and extension would result in the retraction and extension of the robot’s telescopic leg, respectively. The robot was connected to the user at the pelvis via a pelvic harness and at the feet via shoes.

The medial (between-leg) location of the exoskeleton’s legs, also seen in the Honda bodyweight support device [15], and the WPAL [16] offer a number of benefits over the more common outside leg design, allowing (a) a more compact design including a smaller robot hip and user attachment design; (b) the reaction of the swing leg actuation to be transferred directly to the stance leg and, eventually, the ground; and (c) the use of the dual hip motor setup to effectively modulate users’ torso orientation (hip strategy) as well.

Due to the medial (between-leg) design, the robot’s hip joint was located approximately 200 mm distal (below) to the user’s hip joint. The robot’s joints’ range of motion (RoM) were initially estimated from recorded data of normal walking [17,18] and then adjusted for obstacle navigation through kinematic simulations.

The robot hip joint consisted of an active Y rotation in the sagittal plane (hip flexion and extension) and a passive X rotation (hip abduction and adduction) in the frontal plane. The hip joint range of motion was 60° flexion/extension and 16° adduction, but abduction and internal/external rotations were unconstrained. The robot knee flexion/extension RoM was dependent on the user’s leg length. It was designed to accommodate 60° flexion for user heights between 1.55 m and 1.95 m, to address the target range of walking and obstacle navigation scenarios. The robot’s ankle joint consisted of three passive unconstrained DoFs. The 1.55–1.95 m subject height range was accommodated without the need for any adjustments. The total mass of the robot was 11 kg (excluding the offboard power supply and sensor conditioning electronics).

### 2.2. Actuation and Control

**Figure 2:**
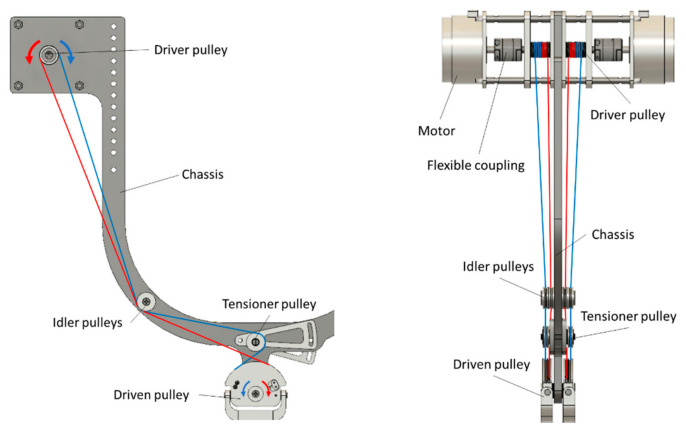
Two independent steel wire pulley systems transmitted torque from the motors to the robot’s hip joints between the user’s legs. The pulley systems also provided a 1:4 gear ratio between the motors and the hip joints.

The robot hip flexion/extension were independently actuated using a separate Maxon EC90 motor (nominal torque: 1.49 Nm; peak torque: 13.1 Nm; mechanical time constant: 7.99 ms; inertia: 5100 gsm^2^; efficiency: 87%) with a steel cable transmission system (Figure 2). This provided a 1:4 gear ratio between the driving pulley at the motor and the driven pulley integrated in the robot’s hip joint. This allowed for a non-continuous peak torque of 27.6 Nm at the hip joint. Idler pulleys were used to route the cable around the curved chassis, allowing the motors and controller boards to be located at the back, outside the movement envelope of the legs.

In this study, to assess the reaction of test subjects to different robot interventions, the exoskeleton was controlled using an open-loop controller, to keep the independent variable (torque magnitude) constant. The robot hip flexion/extension torque was applied constantly during the swing phase of the test leg, simulating modulation of the x position of the heel strike to assist with obstacle navigation. The initiation and termination times of the swing phase were detected using two force sensitive resistors (FSRs) within the sole of each shoe. The swing phase was assumed to start when both FSRs of the swing foot had dropped below a voltage threshold. The robot was tested in tethered mode, where the power supply and the high-level control system were offboard. Tethering allowed the use of high performance actuators and flexible real-time control hardware, providing the capability for rapid prototyping and real-time adjustment of the controller parameters without adding mass to the user.

The exoskeleton uses rotary encoders at the actuators and hip and ankle joints, as well as linear position sensors on the telescopic legs to measure full lower-limb kinematics of the robot and the user in the sagittal plane (Table 1). These sensors further provided safety information and allowed the difference between the hip and actuator angles to be monitored to identify any potential slippage in transmission.

Table 2 compares the peak torque, gear ratio and weight of the system developed in this study with key hip exoskeletons reported in the literature that are used for balance and fall prevention. The exoskeleton developed in this study includes some distinctive features, designed to enable highly repeatable, transparent, and accurate measurements of the effects of different foot-placement modulation schemes in the AP direction on users’ movement and balance. These are: (a) a minimum number of attachment points (3 points) to the body, only at shoes and at the hip for comfort as well as the possibility of accurate measurement of the interaction forces; (b) extremely lightweight leg assembly (approximately 700 g per leg), creating minimal inertia while walking; (c) telescopic legs, enabling only two actuators to be used to bilaterally modulate both foot placements in the AP direction and torso orientation (hip strategy); (d) high back drivability of the actuation system, due to a very low gear ratio (1:4) and minimized friction in all joints; (e) transfer of the reaction of the actuator torque directly to the ground through the contralateral leg, instead of the subject’s hip; and (f) unrestrained range of motion (for the target tasks) at all joints.

## 3. Experimentation

### 3.1. Protocol

**Figure 3:**
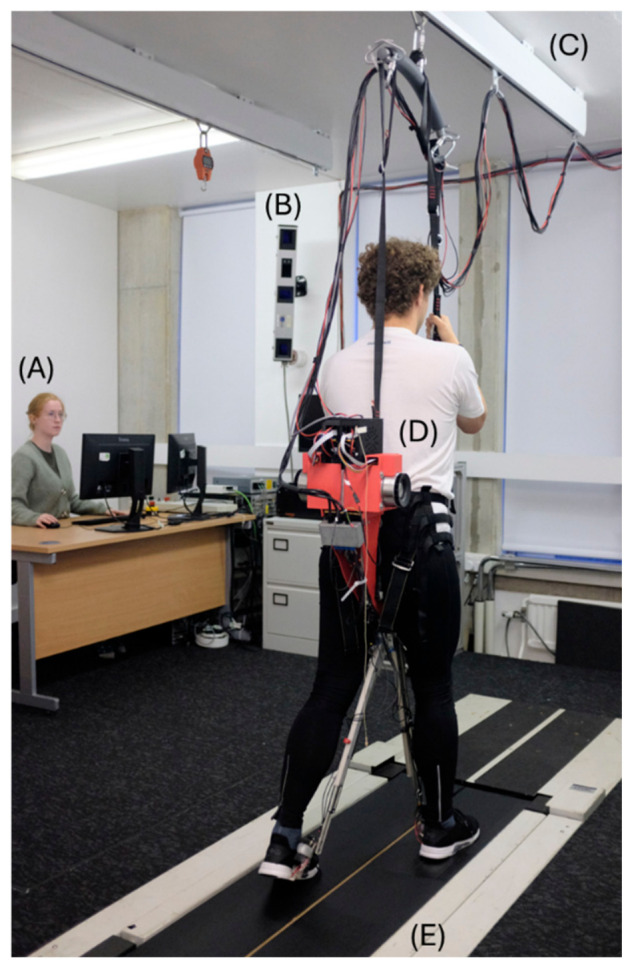
Experiments to test the effectiveness of the robot were conducted with three participants at the HEAD laboratory, University of Bath. (A) Operator and offboard components (PC for control and data collection, power supply, sensor conditioning unit), (B) Codamotion motion capture system, (C) safety harness system, (D) a test subject wearing the developed exoskeleton, and (E) the dual-belt treadmill.

Three healthy adults (one female, two males; mean height: 1.75 m, mean mass: 74.6 kg, mean age: 33 years) participated in the experiment carried out in the HEAD laboratory at the University of Bath (Table 3). The subjects gave informed consent before participating in the experiments. The experimental protocol was approved by the University of Bath Research Ethics Approval Committee for Health (REACH; Reference: EP 22 075). The participants walked on a dual-belt instrumented treadmill at a constant speed of 1 m/s, wearing the exoskeleton (Figure 3). A ceiling-mounted safety harness system was used to ensure safety of the subjects during the tests.

As the participant walked on the treadmill, the exoskeleton was used to apply a force to the swing foot, perpendicular to the orientation of the telescopic leg of the swing leg. To avoid anticipation and/or adaption by the user, the test steps (with force applied to the swing foot) were interspersed randomly between 3 and 7 control steps (no modulation). The controller would count the number of steps and apply a random sequence of torque conditions in test steps only. During a test step, a constant hip torque would be applied throughout the swing phase. Since the negative hip torque reduces the step length and limits the base of support (similar to tripping), it poses a threat to the dynamic balance. As such, through trial and error, the magnitude of the negative torque was capped to 12 Nm to prevent the possibility of a fall.

All three test subjects reported the use of the robot as comfortable and not noticeably hindering their natural motion, although the weight of the device around the hip was noted. This was validated by comparing the mean and SD of the CoM position and velocity in the sagittal plane, stride length and pacing frequency for each subject with and without wearing the exoskeleton (in passive mode), which showed less than 5% difference.

The magnitude of the modulation torque applied at the robot hip in the experiments are shown in Table 4. These conditions were applied in a random order, and each were repeated over 100 times per participant.

### 3.2. Measurements

The control and data acquisition were performed using MATLAB Simulink Desktop Real Time R2022b [26] at a sampling frequency of 100 Hz. The measured signals included the robot hip and ankle angles, robot telescopic legs’ length, test subject’s full body 3D kinematics, treadmill speed, actuators temperature, angle and current, and FSR sensor measurements. The controller output was the target torque/current magnitude, direction, and timing of the torque application.

A Coda optical motion capture system [27] recorded 3D kinematic data of the participants at the same sampling rate. Markers were placed on key bony landmarks of the lower limbs and torso following the VICON Plug in Gait protocol [28]. Although all joint sensor signals were logged, only the force sensitive resistors (FSRs) and hip angle measurements were used by the controller in this study. Swing phase initiation and termination were detected using the FSRs, with swing onset defined as the moment when both FSRs on a given foot dropped below a voltage threshold of 0.4 V. This threshold was determined from initial testing by comparing motion-capture-derived toe-off and heel-strike events with the corresponding FSR signals. Based on these FSR-derived gait events and ensuring that hip angle safety limits were not exceeded, the controller counted the steps, and delivered hip Fl/Ex torques in a randomized sequence.

The measured data were post processed in MATLAB. At the start of each trial, participants performed a standardized calibration movement, enabling manual synchronization of the robot’s onboard sensors with motion capture recordings within a maximum one sample (0.01 s) error. The kinematic data were filtered using a zero lag, fourth-order Butterworth filter with a 15 Hz cutoff frequency, to remove the high frequency noise from marker position data. Zero lag filtering prevented phase distortion, which was essential for the accurate identification of gait events.

Gait events, such as toe off (TO) and heel strike (HS), were identified using Zeni et al.’s [29] protocol. For treadmill walking, the anterior–posterior displacement of the ankle marker relative to the pelvis was used to classify the stance (negative displacement) and swing (positive displacement). Zero crossings between these regions defined toe off (TO) and heel strike (HS). Each gait cycle was segmented from one TO to the subsequent TO of the same foot. Cycles were excluded if TO or HS could not be reliably identified.

Three types of steps were extracted and categorized during post-processing: control, intervention, and post intervention steps. Intervention steps were those in which a torque condition was applied. Control steps were defined as the step immediately preceding an intervention step, representing the gait cycle least influenced by the perturbation. Post intervention steps were the steps immediately following an intervention, capturing the potential after effects of the applied torque and its continuation into subsequent steps. All step classifications were based on the leg receiving the intervention (the “test leg”). Within each cycle, the step type (control, intervention, or post intervention), stride length, and joint kinematics of the test leg were extracted. Stride length was computed as the difference in ankle X position between consecutive TO events.

For each participant and condition, cycles of the same type were grouped and normalized. Time normalization was performed by resampling each cycle to 101 points (0–100% gait cycle). Spatial normalization was achieved by expressing marker trajectories relative to a body-fixed coordinate system with its origin at pelvis position. These procedures enabled direct comparison across cycles and subjects.

For each participant, 800–1000 test steps were collected across eight torque conditions (>100 steps per condition), providing sufficient observations for the mixed-effects modeling of stride length changes.

To evaluate whether stride length varied systematically with the applied hip torque, a linear mixed-effects regression was fitted in SPSS v31 Statistics. This modeling approach was selected because it accommodated repeated measures, unequal numbers of steps per condition, and inter subject variability. Torque magnitude and direction (positive flexion vs. negative extension) were included as fixed effects, while subject and trial were modeled as random intercepts to account for individual baseline differences and within-session correlations. This structure allowed the model to estimate the population level effects of torque while preserving subject-specific variability. Model assumptions (linearity, homoscedasticity, and normality of residuals) were checked using residual plots and Q–Q diagnostics, and no violations requiring model modification were observed.

## 4. Results

### 4.1. Change in Stride Length

**Figure 4:**
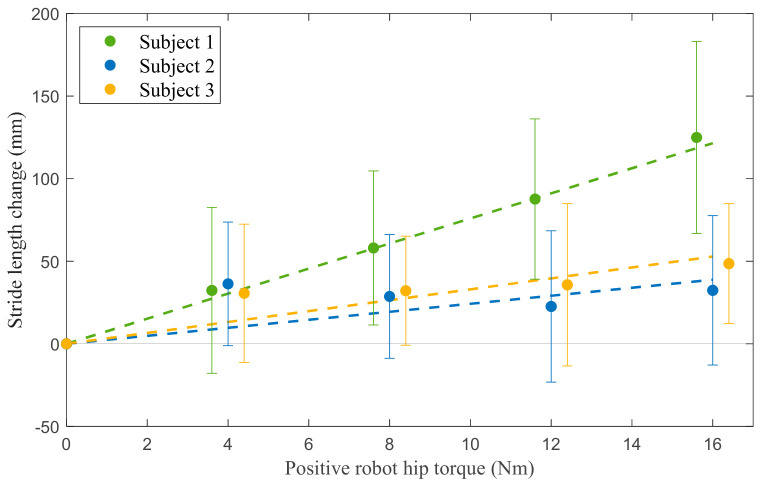
Changes in stride length versus robot hip torque (mean ± SD).

**Figure 5:**
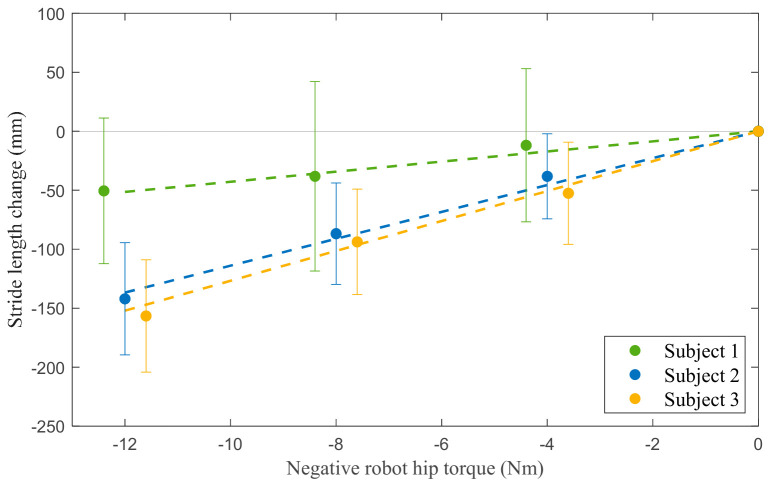
Change in stride length versus the magnitude of negative hip torque (mean ± SD).

Figure 4 and Figure 5 show the mean and SD of the measured changes in stride length of the participants for different robot hip flexion (positive) and extension (negative) torque magnitudes, respectively. The Type III Tests of Fixed Effects confirmed that torque magnitude was a significant predictor of stride length in both flexion and extension conditions (*p* < 0.001; Figure 4 and Figure 5): negative (extension) torque significantly reduced stride length (β = −24.7, SE = 7.8, *p* = 0.002, 95% CI [−40.1, −9.3]), whereas positive (flexion) torque significantly increased stride length (β = 27.9, SE = 2.5, *p* < 0.001, 95% CI [23.1, 32.8]). These results further indicate that while both torque directions significantly influenced stride length, the positive torque effect was estimated with greater precision (narrower confidence interval and smaller SE) compared to the negative torque effect.

The random effects structure revealed substantial between-subject variability in torque responsiveness (Table 5). Subject 1 demonstrated a moderate to strong linear relationship (R^2^ = 0.496) between stride length and positive torque, but a weak relationship with negative torques (R^2^ < 0.1). Conversely, Subjects 2 and 3 demonstrated a weak linear relationship (R^2^ < 0.1) between stride length and positive torque, but a moderate to strong relationship with negative torques (R^2^ = 0.504 and 0.485). These subject-specific patterns indicate that although both torque directions, overall, have shown consistent, positively correlated and statistically significant influence on stride length, the magnitude of these effects (and therefore the resistance of the subject to robot intervention) for the three participants in this study are all interestingly direction-biased.

### 4.2. Change in X-Displacement

**Figure 6:**
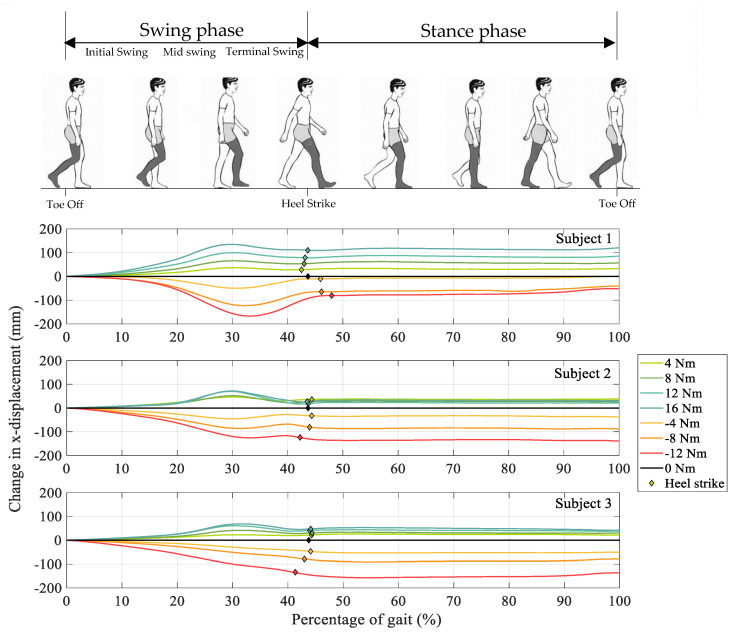
Change in the x-displacement of the test swing ankle over the gait cycle.

Figure 6 shows the difference in the X position (along walking direction—AP) of the swing foot during the gait cycle, between the control and test steps. The intervention torque was applied between 0% and the swing heel strike, shown with a marker on each graph in Figure 6. For each torque magnitude, and at each gait %, the ankle X position of the intervention steps were averaged over the gait cycles and were subtracted from the average ankle X position of the control steps to find their difference (Δx).

Due to the robot architecture, the force applied by the robot on the swing foot is dependent on the hip angle and the telescopic leg length, and is therefore non-linear and time-varying. However, these variations are small compared to the scale of the observed Δx (e.g., at late swing), not correlated with Δx fluctuations, and are inconsistent between test subjects. Therefore, the variations in the force applied on the swing foot do not define the observed changes in Δx and it is hypothesized that these changes are the result of the user’s reaction to the torque.

As can be seen in Figure 6, all subjects had a greater peak Δx for the negative torques compared to the positive torques. Overall, Subject 1 had the greatest peak Δx for both negative and positive torques compared to Subjects 2 and 3. This could potentially be related to Subject 1’s smaller height (shorter legs result in higher force on swing leg for the same robot hip torque), weight (lower leg inertia) or muscle strength in comparison to the other subjects. However, for the negative torques, the change in stride length surprisingly did not follow the same trend, and Subject 1 recorded the least stride length change compared to Subjects 2 and 3.

Except for the negative torques in Subject 3, all subjects showed a clear pattern of correcting the swing foot X position, represented by the negative slope of the graphs after peak Δx. The Δx peaked close to the turning point of the mid-swing to the terminal-swing, between 65% and 85% of the swing phase, and started declining before the HS. For the negative torques, the correction of the swing foot X position was very clear in Subject 1, but less pronounced in Subjects 2 and 3. For the negative torques in Subject 3, the change in Δx was monotonic throughout the swing phase, indicating that Subject 3 did not try strongly to resist the intervention and to correct the position of the swing foot during the swing phase. From the motion planning point of view, Subjects 2 and 3 did not resist negative torques during the same swing phase and let the step-length change be corrected in subsequent steps, while subject 1 strongly tried to contain the effects within the same swing phase.

In Subject 1, the changes in the heel-strike timing, particularly for negative torques was interesting to note. While the stride length decreased with negative correlation to the negative torque magnitudes, interestingly, the swing time increased with positive correlation to the negative torque magnitudes. This is in line with Subject 1’s strategy to oppose the negative torque immediately and can be attributed to a coordinated deceleration pattern across the whole body, providing more time for the localized correction of the foot-placement position before heel strike.

**Figure 7:**
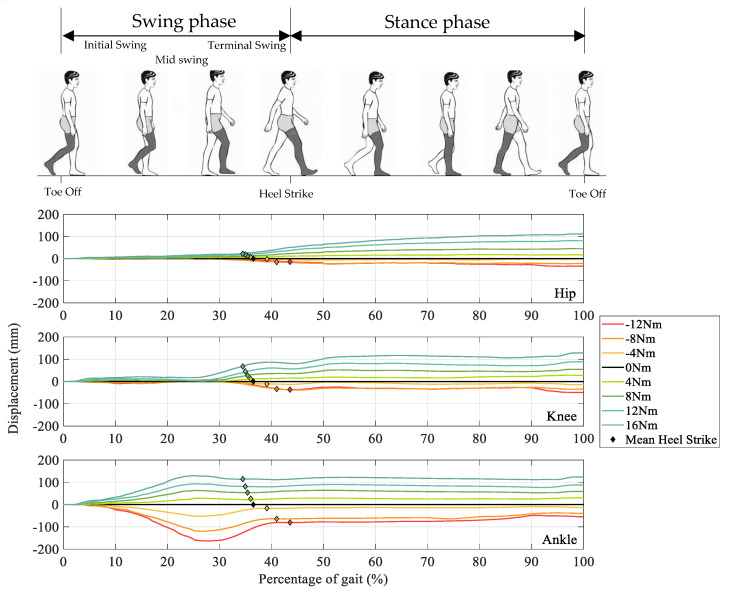
Change in the x-displacement of the test ankle, knee and hip of the test leg in Subject 1.

Figure 7 shows Δx for the ankle, knee and hip of the test leg of Subject 1. Comparing the timing and magnitude of Δx for different joints of the test leg, there is a clear delay in propagation of response to the proximal joints. The majority of Subject 1’s knee Δx has started after the peak ankle Δx, around 30% of the gait cycle, whereas for hip, the majority of the Δx happens after test leg’s heel strike between 40 and 50% of the gait cycle. Such a sequential delayed response in the proximal joints of the test leg enable the modulation of the upper-body and center-of-mass kinematics during the subsequent stance phase to maintain a body-level dynamic and to transit smoothly to the preceding natural walking regime [30,31,32,33,34]. This can be further hypothesized to be the manifestation of the sequential engagement of supra-spinal balance mechanisms, after the engagement of the faster local feedback loops to maintain body-level balance [34,35,36].

## 5. Discussion and Conclusions

The applied hip torques produced systematic direction-biased changes in gait kinematics, with negative (extension) torques exerting a larger influence on stride length and forward ankle displacement in two out of the three test subjects compared with the positive (flexion) torques. Since the biological motor control system optimizes the step length predominantly to maximize stability and efficiency [37], a hypothesis can be that this bias is attributed to the hierarchy of walking efficiency and balancing strategies for this form of perturbation, informed by anthropometric and MSK characteristics.

Subject 1 has a shorter height/leg length and consequently, a shorter natural step length and margin of dynamic stability (MoS). The results of Figure 4 and Figure 5 can be explained by hypothesizing that Subject 1’s smaller MoS in the AP direction means that their dynamic balance is more sensitive to perturbations that limit AP MoS (balance takes priority) and therefore is strongly resisted. Subjects 2 and 3, however, are taller and have longer natural step lengths and MoS in the AP direction. It can be hypothesized that this larger MoS affords them more balance tolerance, resulting in not strongly resisting the step shortening. Instead, the efficiency of walking and energy expenditure have taken priority and they resist step lengthening more strongly to maintain an efficient walking pattern.

The mixed effects analysis showed that torque direction significantly influenced stride length. Modest R^2^ values are typical in human gait studies predominantly due to a significant intra-subject step-to-step variability in gait patterns. Despite this variability, the statistically significant fixed effect estimates demonstrate that hip torque reliably modulated stride length across participants. The magnitude of these changes is practically meaningful for exoskeleton control, indicating that even simple torque interventions can systematically influence gait behavior.

These findings align with previous works showing that mechanical perturbations applied at foot can alter step length, lower-limb movement trajectory, and center of mass motion. The significant differences between the responses of different participants, further highlights the importance of individual movement strategies and user-specific adaptation to mechanical perturbations. Compared with these studies, the present work contributes a novel experimental platform with unique design features, i.e., (a) minimal contact points with user’s body—only at the feet and pelvis; (b) the ability to transfer the reaction of robot intervention forces directly to the ground; and (c) very lightweight low-inertia leg structure allowing for highly unconstrained and natural movements of the user. These features enable us to isolate the effects of a step-length modulation force applied on the swing foot of the test subject without exerting any extraneous dynamic force on subject’s body. Such a transparent and clean interface is essential to accurately measure, analyze, and optimize the effects of various foot-placement modulation schemes on the user’s movements in future studies.

This study was focused on establishing the functionality of the developed exoskeleton, its capability to modulate swing foot-placement location along walking direction (AP), and to assess within-subject variability of the effects of a contact hip torque magnitude on stride length and swing timing. The consistency of the open-loop controller provided a controlled and repeatable perturbation, while the large number (>100) of measurement samples (test steps) per condition provided high within-subject statistical power, allowing reliable assessment of the effects of a desired torque magnitude/direction on each test participant. Designing effective obstacle navigation strategies/controllers, and generalizing the results over the larger population, however, require purpose-designed closed-loop controller architecture, obstacle navigation experiments and a larger pool of test subjects, which are the next steps of this research and are beyond the scope of this study.

The observed kinematic patterns likely arise from a combination of biomechanical constraints, individual movement strategies, and voluntary adaptation to the imposed torques, highlighting the importance of understanding the underlying neuromuscular processes that determine the user’s reaction to the robot’s intervention. Simultaneous measurements of ground reaction forces and center of plantar pressure, full body 3D kinematics and neuromuscular measurements including EMG and neural recordings in future studies can provide more data to investigate these neuromuscular processes and the subject-specific hierarchy of movement/balance strategies.

The insights gained in this study further postulate that body anthropometry, sex, and muscular strength can inform movement and balance strategies and therefore need to be taken into account by the controller.

## Figures and Tables

**Table 1:** Onboard sensors were used to monitor joint positions and trigger robot intervention.

Measurement Parameter	Sensor
Motor encoder	Maxon MILE Encoder (512 CPT, 2 Channels)
Sagittal Hip joint Angle	Rotary strip membrane sensor
Telescopic leg Length	Linear strip membrane sensor
Sagittal Ankle joint Angle	Hall effect sensor
Foot switch (sole of shoe)	Force Sensitive Resistor

**Table 2:** Comparison of design characteristics with hip exoskeletons used for fall prevention.

Characteristic	This Study	[19,20,21]	[11]	[22]	[23,24]	[25]
Peak torque (Nm)	27.6	35	10.8	18	108	40
Gear ratio	4:1	80:1	79:1	9:1	50:1	8:1
Weight (kg)	11 kg total—1.4 kg legs	4.2	-	4.5	4.78	10.8

**Table 3:** Test subject details.

Subject	Gender	Age	Height [m]	Weight [kg]	Mean Step (Stride) Length [m]
S1	F	29	1.63	58	0.66 (1.32)
S2	M	38	1.82	84	0.77 (1.54)
S3	M	32	1.79	82	0.76 (1.52)

**Table 4:** Test parameters to test the effectiveness of the robot in changing the user’s stride length.

Test Parameters	
Positive Torque magnitude (robot hip flexion)	4, 8, 12, 16 Nm
Negative Torque magnitude (robot hip extension)	−4, −8, −12 Nm

**Table 5:** Torque variance.

Subject	R^2^ Linear Value	
	Positive Torques	Negative Torques
S1	0.496	0.048
S2	0.002	0.504
S3	0.024	0.485

## Data Availability

All relevant data is contained within the article. The original contributions presented in the study are included in the article; further inquiries can be directed to the corresponding author.
